# Risk Factors for Infection After Transrectal Prostate Biopsy: A Population-based Register Study

**DOI:** 10.1016/j.euros.2024.06.015

**Published:** 2024-07-13

**Authors:** Joakim Örtegren, Kimia Kohestani, Olof Elvstam, Håkan Janson, Daniel Åberg, Henrik Kjölhede, Gunnar Kahlmeter, Ola Bratt

**Affiliations:** a Department of Urology, Institute of Clinical Sciences, University of Gothenburg, Göteborg, Sweden; b Section of Urology, Department of Surgery, Region Kronoberg, Växjö, Sweden; c Department of Urology, Sahlgrenska University Hospital, Göteborg, Sweden; d Department of Infectious Diseases, Region Kronoberg, Växjö, Sweden; e Department of Translational Medicine, Lund University, Malmö, Sweden; f Department of Clinical Microbiology, Region Kronoberg, Växjö, Sweden; g Regional Office, Region Kronoberg, Växjö, Sweden

**Keywords:** Prostate biopsy, Transrectal, Risk factor, Infection, Register study, Population-based

## Abstract

**Background and objective:**

Infection after transrectal prostate biopsy (TPBx) is a well-known risk. A comprehensive investigation of risk factors may identify measures for safe TPBx as an alternative to a change in biopsy route. The aim of this study was to identify risk factors for infection after TPBx.

**Methods:**

We included all outpatient TPBx cases in Region Kronoberg, Sweden, from January 2010 to December 2019. The primary outcome was post-TPBx infection, defined as prescription of antibiotics indicated for urinary tract infection (UTI) or inpatient care for infection within 30 d. We analysed the following factors in relation to post-TPBx infection: age, diabetes mellitus, prostate cancer diagnosed at index biopsy, previous prostate biopsy, two or more biopsies in the past 24 mo, a positive urine culture, two or more negative urine cultures (UCs) in the past 24 mo, antibiotic treatment grouped as four types, and medication for benign prostatic hyperplasia (BPH). Logistic regression was used to calculate odds ratios (ORs).

**Key findings and limitations:**

Of 5788 TPBx procedures in 4040 patients, 405 (7.0%) led to an infection and 170 (2.9%) to inpatient care for infection. Risk factors for post-TPBx infection (ORs 1.5–2.5) were diabetes mellitus, antibiotic treatment for a UTI, fluoroquinolone treatment, and a positive urine culture. Weaker risk factors (ORs 1.3–1.5) were non-UTI antibiotic treatment, BPH medication, and negative UCs before TPBx.

**Conclusions and clinical implications:**

Our results confirm that diabetes mellitus and previous UTI are risk factors for infection after TPBx. Lower urinary tract symptoms and treatment with any kind of antibiotic were associated with infection, which has not been previously reported.

**Patient summary:**

In a large population-based study from Sweden, we investigated which clinical factors increase the risk of an infection after transrectal prostate biopsy. Our results confirm that diabetes and a previous urinary tract infection are risk factors. We also found two new factors associated with the risk of infection after biopsy: lower urinary tract symptoms and any antibiotic treatment.

## Take Home Message

This large population-based study confirmed diabetes mellitus and previous urinary tract infection as risk factors for infection after transrectal prostate biopsy. We also found that lower urinary tract symptoms and any previous antibiotic treatment were associated with infection after biopsy.

## 1 Introduction

A prostate biopsy is standard for diagnosis of prostate cancer. With more than 2 million performed each year in Europe and the USA [1], prostate biopsy is the most common diagnostic procedure in urology. The European Association of Urology guidelines recommend the transperineal route [2], but the 2023 American Urological Association guidelines conclude that the transrectal and transperineal routes each have advantages and disadvantages, and that it is up to the clinician to choose their preferred route [3].

Observational studies suggest that the transperineal biopsy route is associated with a lower risk of infectious complications in comparison to the transrectal route [4], but the first randomised trials found similar infection rates after transperineal biopsy without and transrectal biopsy with antibiotic prophylaxis [5], [6]. Antibiotic prophylaxis reduces the risk of infection after transrectal prostate biopsy [7], but even with prophylaxis, 3–10% of patients experience an infectious complication [1], [8], [9]. A postbiopsy infection is a burden for both the patient and the health care system [10] and increasing rates over time have been reported [11].

Measures to further reduce the risk of infectious complications after transrectal biopsy include rectal cleansing with povidone-iodine [12], a prebiopsy rectal swab to direct antibiotic prophylaxis [13], modified prophylaxis for patients at risk of microbial resistance [14], [15], [16], and identification of measures to mitigate risk factors.

Diabetes mellitus and previous urinary tract infection (UTI) are established risk factors for postbiopsy infection [17], [18]. For other potential risk factors the results are conflicting. Previous prostate biopsy was associated with postbiopsy infection in an active surveillance protocol [19] but not in a retrospective study [20]. Recent antibiotic treatment was associated with infection in a Turkish study and a Danish study [20], [21] but not in a study that included patients from 13 countries in Europe, Asia, and Africa [8]. Prostate enlargement was associated with infection in a study in a screening setting [22] but not in a clinical setting [8].

The uncertainty about previously reported factors, common in routine urological care, and possibly associated with infection after transrectal biopsy led us to design a large population-based register study to identify significant risk factors. We investigated lower urinary tract symptoms (LUTS) and antibiotics grouped as four types as new potential risk factors for postbiopsy infection.

## 2 Patients and methods

### 2.1 Study design, setting, and participants

The study included all patients who underwent an outpatient transrectal ultrasound-guided prostate biopsy from January 1, 2010 to December 31, 2019 in Region Kronoberg, Sweden. Inpatient biopsy procedures were excluded because these patients usually have a medical condition that affects their management and commonly receive antibiotic treatment rather than prophylaxis. Biopsy procedures in patients with a urine culture sampled from a catheter, urostomy, or nephropyelostomy were excluded as these patients often have resistant bacteria and receive targeted antibiotic treatment.

The standard prebiopsy antibiotic prophylaxis in Region Kronoberg and throughout Sweden [23] during the study period was a single oral dose of 750 mg of ciprofloxacin. Patients with a fluoroquinolone allergy usually received 800/160 mg trimethoprim-sulfamethoxazole. The overall prevalence of fluoroquinolone-resistant bacteria in urine cultures in Region Kronoberg was stable at ∼10% during study period. From January 2015 to 2019, a urine culture before prostate biopsy was clinical routine in Region Kronoberg to identify patients at higher risk of postbiopsy infection. This routine did not reduce the incidence of postbiopsy infection [24] and was thus abandoned in 2022. Rectal cleansing with povidone was not routine during study period.

The standard biopsy procedure during the study period was sampling of 10–14 systematic cores after a periprostatic injection of local anaesthetic. From 2010 to 2018, 344 patients were included in a randomised trial evaluating a biopsy protocol with anterior sampling [25].

### 2.2 Data collection

The electronic medical records (Cambio Cosmic) in Region Kronoberg are comprehensive and include drug prescription and diagnosis and intervention codes for outpatient visits and inpatient episodes. We included outpatient visits coded TKE00 (prostate needle biopsy) or KEB00 (prostate biopsy) according to the NOMESCO Classification of Surgical Procedures.

Urine and blood culture results were retrieved from a database at the Region Kronoberg Region Kronoberg Department of Clinical Microbiology. All results for cultures sampled from October 1, 2008 to January 31, 2020 were included from 2 yr before to 30 d after the biopsy date.

Medical records for patients admitted to hospital within 30 d after biopsy and a discharge ATC code related to UTI or sepsis (see definition below) were reviewed to validate the registration of complications and to identify patients treated at an intensive care unit.

The study was approved by the Swedish Ethical Review Authority (2020-02066).

### 2.3 Potential risk factors

The following potential risk factors were investigated: age at biopsy, diabetes mellitus (E10/E11), prostate cancer (C61) diagnosed at index biopsy, any prostate biopsy in the past 6, 12, or 24 mo, two or more prostate biopsy procedures in the past 24 mo, a positive urine culture in the past 12 or 24 mo, two or more negative urine cultures in the past 24 mo (proxy for noninfectious LUTS), prescription of medication for benign prostatic hyperplasia (BPH) before or within 6 mo after the biopsy (proxy for LUTS), and antibiotic exposure, categorised into the following four groups:

- UTI-antibiotic: treatment with an antibiotic indicated for UTI within 6 wk before biopsy (to capture those who received antibiotics for a positive prebiopsy urine culture or were considered at high risk of infection for other reasons).
- Non-fluoroquinolone UTI-antibiotic: treatment with an antibiotic indicated for UTI excluding fluoroquinolones from 12 mo to 6 wk before biopsy (to capture previous UTI).
- Fluoroquinolone-antibiotic: fluoroquinolone treatment from 6 or 12 mo to 6 wk before biopsy (to capture UTI and fluoroquinolone-resistant rectal flora).
- Non-UTI antibiotic: any other antibiotic treatment from 12 mo to 6 wk before biopsy (to capture nonspecific microbiome alterations and susceptibility to infection).

All urine cultures with growth of *Escherichia coli* were reported as positive, whereas growth of other Enterobacterales or enterococcal species was reported as positive only if >10^8^ cfu/l was detected.

Drug prescriptions were categorised according to their Anatomical Therapeutic Chemical (ATC) code. We defined antibiotic treatment that could be used for UTI as: J01CA (penicillin with extended spectrum), J01DB (first-generation cephalosporin), J01DD (third-generation cephalosporin), J01EA (trimethoprim and derivatives), J01EE (combinations of sulfonamides and trimethoprim), J01MA (fluoroquinolone), J01RA (combination of antibacterials), and J01XE (nitrofuran derivatives). Treatment for BPH was defined as a prescription with ATC code G04C.

### 2.4 Outcome measures

The primary outcome measure was infection within 30 d after biopsy, defined as prescription of an antibiotic indicated for UTI 1–30 d (a prescription on the same day was regarded as prolonged prophylaxis) or hospital admission for UTI or sepsis 0–30 d after the date of prostate biopsy, defined by the diagnosis codes N39 (UTI), N30 (cystitis), T814 (complication after intervention), or A41 (sepsis). The secondary outcome measure was inpatient care for UTI or sepsis 0–30 d after biopsy according to the definition above.

### 2.5 Statistical analysis

Potential risk factors were assessed via univariable logistic regression. Odds ratios (ORs) with 95% confidence intervals (CIs) were calculated. We analysed age as a continuous variable and the other parameters as dichotomous variables. As a sensitivity analysis, the analysis was repeated for the period 2010–2014, when a prebiopsy urine culture was not routinely obtained. Analyses were performed with R v4.3.2, and SPSS v27. The manuscript adheres to the Strengthening the Reporting of Observational Studies in Epidemiology (STROBE) checklist for observational cohort studies.

## 3 Results

**Fig. 1:**
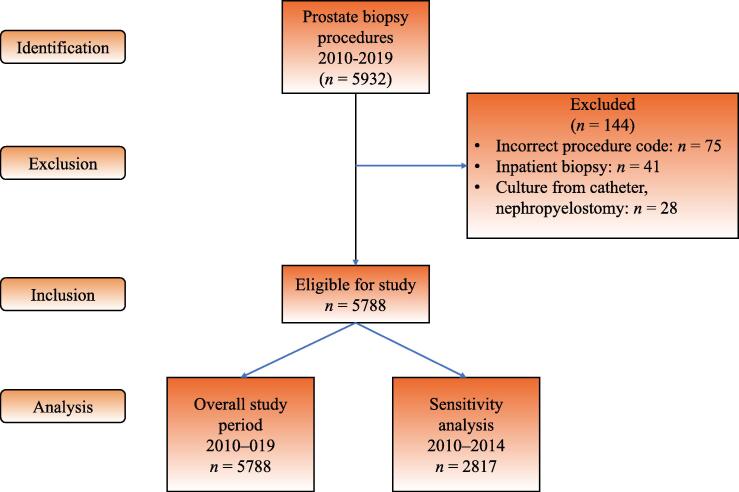
STROBE (Strengthening the Reporting of Observational Studies in Epidemiology) flowchart for the study population.

The study included 5788 prostate biopsy procedures in 4040 patients (Fig. 1). Patient characteristics at the time of biopsy are presented in Table 1. A total of 405 (7.0%) biopsy procedures were followed by a postbiopsy infection within 30 d, of which 170 (2.9% of the biopsy procedures) led to inpatient care. No patient was treated for postbiopsy infection in an intensive care unit.

**Table 1:** Patient characteristics at the time of PB

Parameter and time before PB	Result, *n* (%)	
Age category		
<65 yr	2087 (36.1)	
65–74 yr	2692 (46.5)	
≥75 yr	1009 (17.4)	
	**Yes**	**No**
Diabetes mellitus	584 (10.1)	5204 (89.9)
Prostate cancer	534 (9.2)	5254 (90.8)
UTI antibiotic 6–0 wk	791 (13.7)	4997 (86.3)
Non-FQ UTI antibiotic 1 yr–6 wk	179 (3.1)	5609 (96.9)
FQ antibiotic 6 mo–6 wk	692 (12)	5096 (88)
FQ antibiotic 1 yr–6 wk	918 (15.9)	4870 (84.1)
Non-UTI antibiotic 1 yr–6 wk	446 (7.7)	5342 (92.3)
Positive UC 1 yr–6 wk	264 (4.6)	5524 (95.4)
Positive UC 2 yr–6 wk	357 (6.2)	5431 (93.8)
Previous PB 6–0 mo	581 (10)	5207 (90)
Previous PB 12–0 mo	914 (15.8)	4874 (84.2)
Previous PB 24–0 mo	1349 (23.3)	4439 (76.7)
≥2 PBs within 2 yr	141 (2.4)	5647 (97.6)
BPH medication before or 6 mo after PB	1831 (31.6)	3957 (68.4)
≥2 negative UCs 2 yr–6 wk	1498 (25.9)	4290 (74.1)

FQ = fluoroquinolone; UC = urine culture; PB = prostate biopsy; UTI = urinary tract infection; BPH = benign prostatic hyperplasia.

The analysis identified the following moderately strong risk factors for postbiopsy infection (ORs 1.5–2.5): diabetes mellitus, treatment with a UTI-antibiotic 0–6 wk before biopsy, non-fluoroquinolone UTI antibiotics 1 yr to 6 wk before biopsy, fluoroquinolone treatment 1 yr to 6 wk before biopsy, and positive urine culture in the past 12 or 24 mo (Table 2). Weaker risk factors (ORs 1.3–1.5) were fluoroquinolone treatment 6 mo to 6 wk before biopsy, previous non-UTI antibiotic treatment, BPH medication, and two or more negative urine cultures 2 yr to 6 wk before biopsy (Table 2). The antibiotic types and the number of prescriptions 1 yr to 6 wk before biopsy are listed in Supplementary Table 1. The proportions of patients with a postbiopsy infection by type of previous antibiotic treatment are listed in Supplementary Table 2. Age was of borderline statistical significance (*p* = 0.07). The association between age and postbiopsy infection is illustrated in Supplementary Figure 1.

**Table 2:** Logistic regression results for potential risk factors for an infectious complication within 30 d after transrectal PB

Parameter and time before PB	Odds ratio (95% confidence interval)	
	2010–2019	2010–2014 a
Age	1.01 (0.99–1.02)	1.02 (1.00–1.04)
Diabetes mellitus	1.76 (1.32–2.37)	1.64 (1.06–2.56)
Prostate cancer	0.957 (0.67–1.36)	1.11 (0.72–1.71)
UTI antibiotic 6–0 wk	1.73 (1.34–2.23)	1.51 (1.06–2.16)
Non-FQ UTI antibiotic 1 yr–6 wk	2.51 (1.61–3.90)	0.91 (0.33–2.56)
FQ antibiotic 6 mo–6 wk	1.39 (1.05–1.85)	1.53 (1.06–2.20)
FQ antibiotic 1 yr–6 wk	1.56 (1.20–2.01)	1.37 (0.96–1.94)
Non-UTI antibiotic 1 yr–6 wk	1.44 (1.01–2.05)	1.13 (0.67–1.92)
Positive UC 1 yr–6 wk	1.35 (0.88–2.08)	1.43 (0.68–2.98)
Positive UC 2 yr–6 wk	1.59 (1.11–2.27)	1.22 (0.63–2.36)
Previous PB 6–0 mo	0.87 (0.61–1.23)	0.67 (0.37–1.18)
Previous PB 12–0 mo	0.85 (0.63–1.13)	0.61 (0.39–0.98)
Previous PB 24–0 mo	0.81 (0.63–1.04)	0.75 (0.52–1.10)
≥2 PBs within 2 yr	0.90 (0.46–1.79)	0.81 (0.33–2.02)
BPH medication before or 6 mo after PB	1.34 (1.09–1.65)	1.12 (0.82–1.53)
≥2 negative UCs 2 yr–6 wk	1.32 (1.06–1.64)	1.29 (0.87–1.91)

UTI = urinary tract infection; FQ = fluoroquinolone; UC = urine culture; PB = prostate biopsy; BPH = benign prostatic hyperplasia.

a Sensitivity analysis for the period without routine prebiopsy UC (this was routine in 2015–2019).

Significant risk factors for a postbiopsy infection leading to hospital admission were previous treatment with a non-fluoroquinolone UTI-antibiotic (OR 2.5, 95% CI 1.3–4.7) diabetes mellitus (OR 2.3, 95% CI 1.6–3.3), previous fluoroquinolone treatment, BPH medication, and two or more previous negative urine cultures (ORs 1.4–1.5 for the latter three; Table 3).

**Table 3:** Logistic regression results for potential risk factors for inpatient care for an infectious complication within 30 d after a transrectal PB

Parameter and time before PB	Odds ratio (95% confidence interval)	
	2010–2019	2010–2014 a
Age	1.01 (0.99–1.03)	1.02 (0.99–1.06)
Diabetes mellitus	2.30 (1.56–3.39)	1.80 (0.88–3.68)
Prostate cancer	0.61 (0.32–1.16)	0.74 (0.32–1.73)
UTI antibiotic 6–0 wk	1.31 (0.87–1.97)	0.65 (0.30–1.44)
Non-FQ UTI antibiotic 1 yr–6 wk	2.46 (1.30–4.66)	1.50 (0.35–6.33)
FQ antibiotic 6 mo–6 wk	1.23 (0.79–1.92)	1.24 (0.64–2.40)
FQ antibiotic 1 yr–6 wk	1.49 (1.01–2.20)	1.61 (0.91–2.86)
Non-UTI antibiotic 1 yr–6 wk	1.22 (0.69–2.14)	1.07 (0.42–2.73)
Positive UC 1 yr–6 wk	1.32 (0.69–2.53)	2.23 (0.79–6.28)
Positive UC 2 yr–6 wk	1.27 (0.71–2.26)	1.50 (0.54–4.20)
Previous PB 6–0 mo	1.00 (0.60–1.66)	0.99 (0.42–2.32)
Previous PB 12–0 mo	1.01 (0.66–1.53)	0.75 (0.35–1.58)
Previous PB 24–0 mo	0.99 (0.68–1.41)	1.06 (0.60–1.87)
≥2 PBs within 2 yr	0.96 (0.35–2.64)	1.01 (0.24–4.21)
BPH medication before or 6 mo after PB	1.39 (1.01–1.90)	1.23 (0.73–2.09)
≥2 negative UCs 2 yr–6 wk	1.42 (1.03–1.97)	1.67 (0.90–3.10)

UTI = urinary tract infection; FQ = fluoroquinolone; UC = urine culture; PB = prostate biopsy; BPH = benign prostatic hyperplasia.

a Sensitivity analysis for the period without routine prebiopsy UC (this was routine in 2015–2019).

In the sensitivity analysis of the period without a routine prebiopsy urine culture, statistically significant risk factors for postbiopsy infection were age, diabetes, previous fluoroquinolone treatment, previous prostate biopsy, and treatment with a UTI-antibiotic in the past 6 wk (Table 2).

## 4 Discussion

This large population-based register study confirmed diabetes mellitus and previous UTI as risk factors for infectious complications after transrectal prostate biopsy. The presence of either of these factors increased the odds for postbiopsy infection and inpatient care by approximately twofold.

We found that a prescription for a non-UTI antibiotic in the past year was associated with postbiopsy infection. To the best of our knowledge, antibiotic exposure categorised into different groups has not previously been described as a risk factor for postbiopsy infection. Possible explanations are that non-UTI antibiotics lead to fluoroquinolone-resistant *E. coli* via either mutation or selection. At odds with this explanation is a recent study in Sweden in which antibiotic treatment 1 yr before biopsy was not associated with fluoroquinolone-resistant *E. coli* in the rectum [26].

We are also the first to report that men on pharmacological treatment for BPH and men who had two or more previous negative urine cultures were more likely to experience postbiopsy infection. It is likely that these findings reflect LUTS as the actual risk factor associated with postbiopsy infection rather than the BPH treatment. This is in line with a recent report from the Göteborg-2 prostate cancer screening trial [27], in which only 1.5% of the biopsied men had a febrile infection. The screening trial invited men aged 50–60 yr from the general population, in which LUTS prevalence is lower than in the older cohort in our study, many of whom may have initially contacted primary care because of LUTS caused by benign prostatic enlargement, which in turn increased prostate-specific antigen levels, prompting a prostate biopsy. LUTS may be associated with postvoid residual urine and thereby increase the risk of UTI.

Our finding that any antibiotic treatment for UTI increased the risk of postbiopsy infection confirms results from an earlier study [28]. A previous UTI may have led to asymptomatic bacteriuria or residual bacteria in the prostate that predispose the patient to a clinical infection after prostate biopsy.

Previous prostate biopsy has been reported as a risk factor for postbiopsy infection [19] but not even receipt of two or more previous biopsy procedures was associated with higher risk in our study.

The sensitivity analysis for the period without a routine prebiopsy urine culture confirmed the findings from the main analysis, except that UTI-antibiotic treatment in the past 6 wk was associated with postbiopsy infection in the main analysis but not in the sensitivity analysis. An explanation for this may be that the clinical routine meant that some men with a positive prebiopsy urine culture were treated with a fluoroquinolone before biopsy and then developed a fluoroquinolone-resistant rectal bacterial flora but still received fluoroquinolone prophylaxis. It is unlikely that an overall increase in bacterial fluoroquinolone resistance was the cause, as the prevalence of resistant *E. coli* in urine cultures was stable at ∼10% throughout the study period [29].

Our results suggest that not only men with diabetes mellitus or a previous UTI but also those with LUTS or any antibiotic treatment in the past year are at higher risk of postbiopsy infection. These men should be informed about their higher risk and managed accordingly. If the transrectal biopsy route is preferred, they should be offered a rectal povidone-iodine preparation [30], a rectal swab to identity resistant bacteria, or antibiotic treatment rather than a short prophylaxis course. An attractive alternative is the transperineal biopsy route, probably with antibiotic prophylaxis.

Strengths of our study are the large, population-based sample and the use of reliable registers. Weaknesses include the routine prebiopsy urine culture in the later part of the study period, which may have affected the risk of a postbiopsy infection in some patient groups. Moreover, a few patients may have received medical care for an infection outside Region Kronoberg, but it is unlikely that any missed data of this type affected the results.

## 5 Conclusions

This population-based study confirmed diabetes mellitus and previous UTI as risk factors for an infectious complication after transrectal prostate biopsy. Two new potential risk factors were identified: previous non-UTI antibiotic use and LUTS. We are currently investigating the latter findings in a nationwide register study. An assessment of individual risk factors for postbiopsy infection followed by specific risk-reducing measures should be recommended before prostate biopsy via any route.

  ***Author contributions***: Joakim Örtegren had full access to all the data in the study and takes responsibility for the integrity of the data and the accuracy of the data analysis.

  *Study concept and design*: Örtegren, Kjölhede, Bratt, Janson, Kahlmeter, Åberg, Kohestani.

*Acquisition of data*: Örtegren, Kahlmeter, Åberg.

*Analysis and interpretation of data*: Örtegren, Åberg, Bratt, Kjölhede, Elvstam.

*Drafting of the manuscript*: Örtegren, Kohestani, Kjölhede, Bratt, Åberg, Kahlmeter, Janson, Elvstam.

*Critical revision of the manuscript for important intellectual content*: Örtegren, Kohestani, Kjölhede, Bratt, Åberg, Kahlmeter, Janson, Elvstam.

*Statistical analysis*: Örtegren, Åberg, Bratt, Kjölhede, Elvstam.

*Obtaining funding*: Örtegren.

*Administrative, technical, or material support*: None.

*Supervision*: None.

*Other*: None.

  ***Financial disclosures:*** Joakim Örtegren certifies that all conflicts of interest, including specific financial interests and relationships and affiliations relevant to the subject matter or materials discussed in the manuscript (eg, employment/affiliation, grants or funding, consultancies, honoraria, stock ownership or options, expert testimony, royalties, or patents filed, received, or pending), are the following: None.

  ***Funding/Support and role of the sponsor*:** This research was supported by grants from Cancerstiftelsen Kronoberg and by grants from the Swedish Government under an ALF agreement between the Swedish government and county councils (ALFGBG-873181). The sponsors played no direct role in the study.
