# Supplementary material for: Risk Factors for Infection After Transrectal Prostate Biopsy: A Population-based Register Study
Source: Eur Urol Open Sci. 2024 Jul 13;67:1–6. doi: 10.1016/j.euros.2024.06.015 (PMC11298891; doi:10.1016/j.euros.2024.06.015)
Supplement: Supplementary Data 1 [file mmc1.docx]

| *Supplementary Table S1.*  Prescription of antibiotic treatments 6 weeks to 1 year prior to index prostate biopsy | | | | |  |  | | |
| --- | --- | --- | --- | --- | --- | --- | --- | --- |
|  | | | | | | | |  |
| **ATC** | **2010–2014** | **2015–2019** | **Prescriptions** |  | | |  |  |
| J01AA | 168 | 105 | 273 | Tetracyclines | | |  |  |
| J01CA | 71 | 152 | 223 | PC extended spectrum | | |  |  |
| J01CE | 156 | 131 | 287 | Beta-lactamase sensitive PC | | |  |  |
| J01CF | 91 | 105 | 196 | Beta-lactamase resistant PC | | |  |  |
| J01CR | 3 | 4 | 7 | Combinations PC and beta-lactam | | |  |  |
| J01DB | 16 | 12 | 28 | First-generation cephalosporins | | |  |  |
| J01DD | 15 | 2 | 17 | Third-generation cephalosporins | | |  |  |
| J01DH | 1 | 0 | 1 | Carbapenems | | |  |  |
| J01EA | 12 | 3 | 15 | Trimethorprim and derivatives | | |  |  |
| J01EE | 33 | 39 | 72 | Sulfonamid and trimethorprim | | |  |  |
| J01FA | 8 | 10 | 18 | Macrolides | | |  |  |
| J01FF | 33 | 26 | 59 | Lincosamids | | |  |  |
| J01MA | 653 | 498 | 1151 | Fluoroquinolones | | |  |  |
| J01XE | 22 | 58 | 80 | Nitrofuran derivates | | |  |  |
| J01XX | 1 | 2 | 3 | Other antibacterials | | |  |  |
| **Total** | **1283** | **1147** | **2430** |  | | |  |  |

Abbreviations: ATC = Anatomic Therapeutic Chemical classification system, PC = penicillin,
