# Supplementary material for: Risk Factors for Infection After Transrectal Prostate Biopsy: A Population-based Register Study
Source: Eur Urol Open Sci. 2024 Jul 13;67:1–6. doi: 10.1016/j.euros.2024.06.015 (PMC11298891; doi:10.1016/j.euros.2024.06.015)
Supplement: Supplementary Data 2 [file mmc2.docx]

| **Antibiotic treatment** | **Post-biopsy infection** |
| --- | --- |
| Fluoroquinolone (n=918) | 9.2% |
| Non-fluoroquinolone UTI antibiotic (n=179) | 14.0% |
| Non-UTI antibiotic (n=446) | 8.5% |
| No antibiotic treatment (n=4,245) | 6.1% |

*Supplement Table S2*

Previous antibiotic treatments 1 year to 6 weeks before transrectal prostate biopsy and proportions with a post-biopsy infection.

UTI = Urinary Tract Infection. Non-fluoroquinolones UTI antibiotics does not include fluoroquinolones. Non-UTI antibiotic treatment does not include fluoroquinolone or Other UTI.
