# Supplementary material for: Risk Factors for Infection After Transrectal Prostate Biopsy: A Population-based Register Study
Source: Eur Urol Open Sci. 2024 Jul 13;67:1–6. doi: 10.1016/j.euros.2024.06.015 (PMC11298891; doi:10.1016/j.euros.2024.06.015)
Supplement: Supplementary Data 3 [file mmc3.docx]

*Supplementary figure S1*

The proportion of patients with a post-biopsy infection stratified by age at biopsy.
